# Drug Reaction with Eosinophilia and Systemic Symptoms (DRESS) syndrome associated with cefotaxime and clindamycin use in a 6 year-old boy: a case report

**DOI:** 10.11604/pamj.2017.28.218.10828

**Published:** 2017-11-09

**Authors:** Burcu Karakayalı, Ahmet Sami Yazar, Deniz Çakir, Aysen Cetemen, Mandana Kariminikoo, Burak Deliloglu, Sirin Guven, Ismail Islek

**Affiliations:** 1 Healthy Sciences University, Umraniye Training and Research Hospital, Department of Pediatrics, Istanbul, Turkey

**Keywords:** DRESS syndrome, cefotaxime, clindamycin, children

## Abstract

Drug reaction with eosinophilia and systemic symptoms (DRESS) syndrome is a rare and potentially life-threatening idiosyncratic drug reaction. It presents with extensive rash, fever, lymphadenopathy, hematologic abnormalities (eosinophilia and/or atypical lymphocytosis) and internal organ involvement. It has been described in association with more than 50 drugs. To the best of our knowledge neither cefotaxime nor clindamycin has been previously reported to induce DRESS syndrome in children. Clindamycin was reported only in adults as a cause of DRESS syndrome in the literature. In this report, we aimed to present a child with DRESS syndrome that developed after cefotaxime and clindamycin treatment. A 6-year-old boy was diagnosed with the left lower lobe pneumonia and pleural effusion. Parenteral cefotaxime and clindamycin were then started, after which the patient improved clinically and was discharged 7 days later with oral amoxicillin clavulanate treatment. After four days he was readmitted to the hospital with fever and cough. Chest X-ray revealed left lower lobe pneumonia and pleural effusion. We considered that the pneumonia was unresponsive to oral antibiotic treatment, and therefore parenteral cefotaxime and clindamycin were re-administered. As a result, his clinical and radiological findings were improved within 10 days. On the 12^th^ of day of hospitalization, the body temperature has risen to 39°C, which we considered to be caused by antibiotics and stopped antibiotic treatment. At the same day he developed generalized maculopapular erythematous rash, which was considered an allergic reaction secondary to antibiotics. Despite the antihistaminic drug administration, the clinical status quickly deteriorated with generalized edema, lymphadenopathies and hepatosplenomegaly. Laboratory tests revealed a white blood cell count of 4300/μl, a lymphocyte count of 1300/μl, a hemoglobin level of 11.2 gr/dl, a platelet count of 120.000/μl, an eosinophilia ratio of 10% on peripheral blood smear, a C-reactive protein level of 20 mg/dl, a procalcitonin level of 23.94 ng/ml and an erythrocyte sedimentation rate of 48 mm/h. Anti nuclear antibody, anti-double stranded DNA, the serologic tests for Epstein Bar virus, herpes simplex virus, parvovirus, mycoplasma, toxoplasmosis, rubella, cytomegalovirus were all found negative. Bone marrow aspiration was consistent with an autoimmune reaction. An echocardiographic examination was normal. Thoracic tomography revealed multiple enlarged axillary, supraclavicular and anterior mediastinal lymph nodes. As the patient met 8 out of 9 RegiSCAR criteria for the diagnosis of DRESS, we started pulse methyl prednisolone (30 mg/kg/day) for three days followed by 2 mg/kg/day. On the 2nd day fever resolved and cutaneous rash and edema improved. Ten days after developing eruptions the patient was discharged. To our knowledge, we report the first pediatric case of DRESS syndrome following treatment with cefotaxime and clindamycin. Pediatricians should be aware of this potential complication associated with these commonly prescribed antibiotics.

## Introduction

Drug reaction with eosinophilia and systemic symptoms (DRESS) syndrome is a rare and potentially life-threatening idiosyncratic drug reaction. It presents with extensive rash, fever, lymphadenopathy, hematologic abnormalities (eosinophilia and/or atypical lymphocytosis) and internal organ involvement. The incidence of this syndrome ranges from 1/1,000 to 1/10,000 after drug exposure. Adults are more commonly affected than children [1]. Approximately 50 drugs (sulfa derivates, antidepressants, nonsteroidal anti-inflammatory drugs) may induce DRESS syndrome, but it is most commonly induced by anticonvulsants (phenytoin, phenobarbital, carbamazepine) and antibiotics [1,2]. It has been described in association with more than 50 drugs. To the best of our knowledge neither cefotaxime nor clindamycin have been previously reported to cause DRESS syndrome in children. Clindamycin was reported only in adults as an agent causing DRESS syndrome in the literature [1-4]. We present a child with DRESS syndrome presenting 21 days after starting cefotaxime and clindamycin treatment.

## Patient and observation

**Figure 1:**
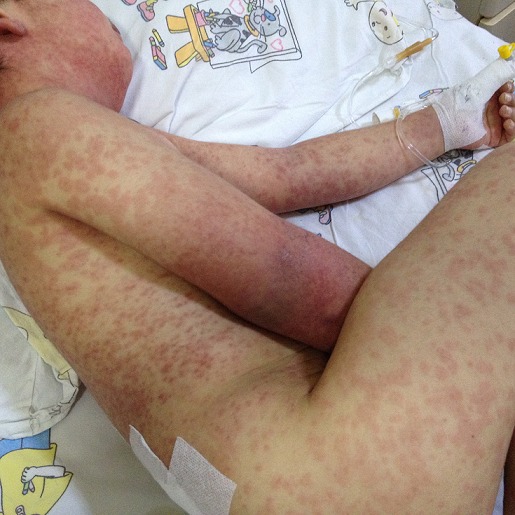
Severe cutaneous rashes and edema

A 6-year-old boy was diagnosed with the left lower lobe pneumonia and pleural effusion and was put on parenteral cefotaxime and clindamycin treatment. He then improved clinically and was discharged 7 days later with oral amoxicillin clavulanate treatment. After four days, however, he was readmitted to the hospital with fever and cough. A chest X-ray revealed left lower lobe pneumonia and pleural effusion. As a case of complicated pneumonia was considered, we started parenteral cefotaxime and clindamycin treatment, 10 days after which his clinical and radiological findings were improved. At the 12^th^ of day of hospitalization, however, he developed at 39°C, which we considered as a side effect of antibiotics. Therefore, antibiotic treatment was stopped. At the same day, he also developed a generalized maculopapular erythematous rash, which was considered an allergic reaction secondary to antibiotics (Figure 1).

Despite the administration of antihistaminic drugs, his clinical status quickly deteriorated with generalized edema, lymphadenopathies and hepatosplenomegaly. Laboratory tests showed a white blood cell count of 4,300/mm^3^, a lymphocyte count of 1,300/μl, a hemoglobin level of 11.2 gr/dl, a platelet count of 120,000/μl, an eosinophil ratio of 10% on peripheral blood smear, a C-reactive protein level of 20 mg/dl, a procalcitonin level of 23.94 ng/ml and an erythrocyte sedimentation rate of 48 mm/h. Anti-nuclear antibody, anti-double strain DNA, the serologic tests for Epstein Bar virus, herpes simplex virus, parvovirus, mycoplasma, toxoplasmosis, rubella, cytomegalovirus were all found negative. Bone marrow aspiration was consistent with an autoimmune reaction. An echocardiographic examination was found normal. A Thoracic computerized tomography revealed multiple enlarged axillary, supraclavicular and anterior mediastinal lymph nodes. As the patient met 8 out of 9 RegiSCAR criteria for the diagnosis of DRESS, pulse methyl prednisolone (30 mg/kg/day) was administered for three days followed by 2mg/kg/day. On the 2nd day fever resolved and cutaneous rash and edema improved. He was discharged ten days after the appearance of eruptions. The patient’s father gave consent for publication of his son’s pictures.

## Discussion

DRESS syndrome was first defined in 1996 by Bocquet et al [5]. It is characterized by fever, cutaneous eruption, internal organ involvement and hematologic abnormalities within 1-8 weeks after the exposure of the suspected drug [5,6]. Anticonvulsants such as carbamazepine, lamotrigine, phenobarbital, phenytoin and allopurinol are the most common causes of DRESS syndrome [1]. In a series of 172 cases of DRESS syndrome carbamazepine accounted for the greatest percentage of cases (27%), followed by allopurinol (11%), lamotrigine (6%), phenobarbital (6%), sulfasalazine (6%) [1].

The pathogenesis of DRESS syndrome has not been elucidated. Different mechanisms have been proposed, including detoxification defects leading to reactive metabolite formation and subsequent immunological reactions, slow acetylation and reactivation of Human Herpes Virus (HHV-6-7) and EBV [1]. Severe DRESS syndrome has also been reported following infection with or reactivation of HHV-6 [7]. CMV and paramyxovirus have been reported as the other viral agents associated with DRESS syndrome. Although it is thought that there is a genetic predisposition to adverse reactions, no genetic factor responsible for the syndrome has been identified yet [1].

Three different sets of criteria are used to diagnose of DRESS syndrome: RegiSCAR criteria, Bocquet’s criteria and Japanese consensus group to diagnose DIHS [5,6]. RegiSCAR criteria include at least 3 of the following 7 characteristics: 1) skin eruption; 2) fever > 38°C; 3) lymphadenopathy involving at least 2 sites; 4) involvement of at least 1 internal organ; 5) lymphocytosis (> 4000/mm^3^) or lymphocytopenia (< 1500/mm^3^); 6) blood eosinophilia (> %10 or 700/mm^3^) and 7) thrombocytopenia (< 120.000/mm3 ) [8]. Three of the four main criteria (fever > 38°C, lymphadenopathy at least 2 sites, involvement of at least 1 internal organ and blood abnormalities) are required for the diagnosis of DRESS syndrome. Additional criteria are hospitalization and drug induced reaction [6, 8, 9]. Our patient met 6 of 7 RegiSCAR criteria and he also had two additional criterias ( hospitalization and drug induced reaction).

The reported mortality of the syndrome ranges between 10% and 40%. Liver damage secondary to eosinophilic infiltration is the most important cause of mortality. There are no consensus guidelines for the management of patients with DRESS syndrome. The most important steps for a proper management include the recognition of the syndrome and immediate discontinuation of the offending drug. The French Society of Dermatology recommends the use of systemic corticosteroids (prednisone 1 mg/kg/day) and intravenous immunoglobulin (2 gr/kg) for five days especially in patients with life threatening internal organ involvement, such as in renal or respiratory failure [10]. Pulse methyl prednisolone treatment was also reportedly administered in a pediatric case of DRESS syndrome secondary to anticonvulsant use [11,12]. Gancyclovir has been suggested in patients with severe signs and the confirmation of a major viral reactivation of HHV-6 [10].

## Conclusion

The diagnosis of the DRESS syndrome should be highly suspected in patients with fever, skin rash, liver involvement, hypereosinophilia and lymphadenopathy that develop during the use of a culprit drug. We report the first case of DRESS syndrome associated with cefotaxime and clindamycin exposure in a 6-year-old boy. Early recognition of the signs of the DRESS syndrome and immediate cessation of the suspected drug are the most important steps for an appropriate management of the syndrome. Pediatricians should be careful about this potential complication associated with these commonly prescribed antibiotics in pediatric practice.

## Competing interests

The authors declare no competing interests.

## References

[1] Cacoub P, Musette P, Descamps V, Meyer O, Speirs C, Finzi L, Roujeau JC (2011). The DRESS syndrome: a literature review. Am J Med..

[2] Walsh SA, Creamer D (2011). Drug reaction with eosinophilia and systemic symptoms (DRESS): a clinical update and review of current thinking. Clin Exp Dermatol..

[3] Miller Quidley A, Bookstaver PB, Gainey AB, Gainey MD (2012). Fatal clindamycin-induced drug rash with eosinophilia and systemic symptoms (DRESS) syndrome. Pharmacotherapy..

[4] Tian D, Mohan RJ, Stallings G (2010). Drug rash with eosinophilia and systemic symptoms syndrome associated with clindamycin. Am J Med..

[5] Bocquet H, Bagot M, Roujeau JC (1996). Drug-induced pseudolymphoma and drug hypersensitivity syndrome (Drug Rash with Eosinophilia and Systemic Symptoms: DRESS). Semin Cutan Med Surg..

[6] Kim DH, Koh YI (2014). Comparison of diagnostic criteria and determination of prognostic factors for drug reaction with eosinophilia and systemic symptoms syndrome. Allergy Asthma Immunol Res..

[7] Ichiche M, Kiesch N, De Bels D (2003). DRESS syndrome associated with HHV-6 reactivation. Eur J Intern Med..

[8] Kardaun SH, Sidoroff A, Valeyrie-Allanore L, Halevy S, Davidovici BB, Mockenhaupt M, Roujeau JC (2007). Variability in the clinical pattern of cutaneous side-effects of drugs with systemic symptoms: does a DRESS syndrome really exist?. Br J Dermatol..

[9] Kardaun SH, Sekula P, Valeyrie-Allanore L, Liss Y, Chu CY, Creamer D, Sidoroff A, Naldi L, Mockenhaupt M, Roujeau JC, RegiSCAR study group (2013). Drug reaction with eosinophilia and systemic symptoms (DRESS): an original multisystem adverse drug reaction. Results from the prospective RegiSCARstudy. Br J Dermatol..

[10] Descamps V, Ben Said B, Sassolas B, Truchetet F, Avenel-Audran M, Girardin P, Guinnepain MT, Mathelier-Fusade P, Assier H, Milpied B, Modiano P, Lebrun-Vignes B, Barbaud A, groupe Toxidermies de la Société Française de dermatologie (2010). Management of drug reaction with eosinophilia and systemic symptoms (DRESS). Ann Dermatol Venereol..

[11] Kocaoglu C, Cilasun C, Solak ES, Kurtipek GS, Arslan S (2013). Successful treatment of antiepileptic drug-induced DRESS syndrome with Pulse Methylprednisolone. Case Rep Pediatr..

[12] El omairi N, Abourazzak S, Chaouki S, Atmani S, Hida M (2014). Drug reaction with Eosinophilia and Systemic Symptom (DRESS) induced by carbamazepine: a case report and literature review. Pan Afr Med J..

